# Limitations of Immunotherapy in Cancer

**DOI:** 10.7759/cureus.30856

**Published:** 2022-10-29

**Authors:** Sanya Gupta, Samarth Shukla

**Affiliations:** 1 Department of Pathology, Jawaharlal Nehru Medical College, Datta Meghe Institute of Medical Sciences, Wardha, IND

**Keywords:** cost efficacy, cancer, tumour, immunotoxicity, cancer immunotherapy, limitations

## Abstract

Despite primary advancements in medication, cancer continues to be one of the main reasons for death globally. Techniques for immunotherapy against malignant improvement are rising as fantastic treatments for this setback. The rapid development of modern immunotherapeutics has inspired a decrease in stubborn malignancies and led to an emotional improvement in patient endurance. However, a giant barrier to these ongoing treatment modalities is an anomaly in clinical response, specifically articulated amongst targeted spot inhibition, chimeric antigen receptor (CAR) T-cell therapy, oncolytic infections, and recombinant cytokines emerging since sickness cells use a variety of structures to avert immunosurveillance. Other challenges encompass the lack of ability to predict the efficacy of the therapy and the response of the patient, the want for additional biomarkers, the improvement of immunity in opposition to malignant boom immunotherapies, the absence of medical assessment plans that are up-to-date to decide sufficiency, and high therapy costs. The therapy for middle-stage malignant growth and the top five difficulties, setbacks, and drawbacks of immunotherapy are examined in this audit.

## Introduction and background

The need for more advanced treatment approaches is pressing, given that there were 9.6 million fatalities and 18.1 million new instances of malignant growth disorder worldwide in 2018. Chemotherapy, radiation, and medical procedures have long been the standard forms of anticancer therapy. While many of these therapies have significantly aided in eliminating serious tumours, the recurrence of disease is still a problem frequently encountered because of persisting cancerous cells and growing metastases. As a result, methods of elective therapy to cope with killing the safe growth cells are necessary [1]. Over the past ten years, we have witnessed several progressive immunotherapies being approved for use in medical settings to treat ill patients. Examples of these immunotherapies include the primary disease immunisation, sipuleucel-T, for advanced prostate cancer; immune checkpoint inhibitors, such as ipilimumab, pembrolizumab, and nivolumab for the treatment of advanced melanoma and other serious diseases; oncolytic infection talimogene laherparepvec (T-Vec) for melanoma; and a bispecific malignant growth coordinated T-cell engager, blinatumomab. These immunotherapies collectively have a remarkable impact on clinical outcomes [2]. Two examples of developments altering the ideal models of clinical disease care are the development of specified spot inhibitors against metastatic melanoma and assenting T-cell treatment with chimeric antigen receptor T-cells (CARTs) against B-cell-determined leukaemias and lymphomas [3]. Despite these advancements, there are still challenges in the field of malignant growth immunotherapy. These include the inability to predict patient response and therapy viability, the need for additional biomarkers, the improvement in disease immunotherapy protection, the lack of streamlined clinical review strategies to determine adequacy, and high treatment costs. Future developments in disease immunotherapy are expected to endure and resolve a sizable number of these issues. Expected outcomes include more targeted therapies, the development of personalised biomarker profiles, drug combination therapies that will improve efficacy and lessen toxicity, and immune-preventive systems that will lower the frequency and repetition of malignant growth and the associated treatment costs [4]. People's clinical outcomes have improved due to the shift in focus from the direct targeting of the malignant growing cell to the excitation of the anti-cancer reaction. Still, this shift also brings with it new problems. Even while checkpoint inhibitors (CPIs), growth vaccines, and adoptive cellular therapy (ACT) or a combination of these have generally objective response rates (ORR), response rates to immunotherapy vary greatly amongst cancer subtypes, primarily depending on their immunogenicity [5].

Methodology

The following review article has been written through extensive research from the pre-existing literature as available in the referred journals and papers, the majority of which are from PubMed, Google Scholar, and Elsevier, including keywords immunotherapy, limitations, tumour suppressive microenvironment, cancer, immunology, tumours, metastatic carcinoma and clinical trials for cancer. Priority is given to text from under five years.

## Review

1. Unpredictable efficacy

The requirement to develop expertise and be consistently effective in a larger population of patients and malignant growth types is an effective testing method for cancerous immunotherapies. Malignant growth immunotherapies have produced emotional results in specific individuals, demonstrating the viability of reestablishing anti-tumour resistance surveillance. However, until now, many immunotherapy treatments have only been effective in a small subset of malignant growths and typically in a small proportion of patients with those diseases [6]. Several explanations for the variable response to disease immunotherapies have been put forth, including the need to identify additional biomarkers and pathways for malignant growth, cancer heterogeneity, variations in malignant growth type and stage, therapy history, and the disease's underlying immunosuppressive mechanisms. Therapies that aim to block malignant development pathways or single atomic changes have only modestly improved tumour durability. This approach, dubbed "reductionist," [7] might be enhanced by controlling medication combinations that concentrate on various disease processes and transformations. Additionally, many changes identified in human growth don't occur consistently across many patients. Immunotherapies coordinated at sub-atomic alterations need indeed be modified and patient-explicit to be more efficacious [8].

The long-standing use of conventional chemotherapy as first-line disease treatment may impede superior survivability with malignant growth immunotherapy. Disease immunotherapies are typically administered to individuals whose immune systems have been impaired by a recent illness and previous treatments because they have not yet been extensively demonstrated as first-line therapy. Higher survival rates may be achieved in more patients on the off chance that personalised malignant growth immunotherapies were previously managed to reestablish a potent anti-tumour reaction. At the same time, the immune system can still recover. The ability of disease immunotherapies to reestablish antitumour-resistant capability under these circumstances is testing [9].

2. Determining the dominant drivers of cancer immunity

Genomic instability, as proven through the microsatellite instability (MSI) or tumour mutational burden (TMB) status, presents a mechanism for developing precise antigenicity for a malignant tumour via means of imparting the host's immune system with something overt and foreign (non-self) to lock onto a cancer-associated antigen to the major histocompatibility complex (MHC) [10]. Clonal transformations, which arise early while oncogenesis starts and have an effect on all disease cells, are commonly much more likely to bring about a competitive, much aggressive enemy of malignant growth T-cellular reaction than later branch transformations, which might be restrained to a selected subset of the disease cells (subclonal changes) [11]. For the malignant improvement insusceptibility cycle to begin (step 1), disorder antigens, specifically neo-antigens, need to show this invulnerable acknowledgement sign [12]. In mild early statistics in metastatic bladder cancer, non-small cell lung cancer (NSCLC), malignant colon cancer, and numerous different malignancies depicting the same, growths with pretty excessive TMB, of which MSI addresses the maximum part of the TMB continuum, these all respond well to CPIs [13]. The question whether excessive TMB cancers from intensely elevated conditions like NSCLC finally get hold of progressed endurance advantage from CPI treatments remains, however, for the reason that triggered growths undergo resistant alterations with advancing lack of heterozygosity (LOH) within the human leukocyte antigen (HLA) locus. Late outcomes from randomised Phase III trial in current NSCLC, together with Checkmate-227 and MYSTIC, recommend that excessive TMB won't be as powerful at predicting the patience gain from a mixture of Programmed Death-1 (PD-1) and Cytotoxic T- Lymphocyte-associated Antigen 4 (CTLA-4) inhibitors.

It is unknown if HLA-LOH confuses the interpretation of those perplexing indicators and whether or not it must be considered whilst describing an immunogenic growth fuelled with the aid of using level 1 of the ailment resistance cycle [14]. The ability of T cells to recognise neoantigens produced by alterations would certainly be impacted by the loss of antigen display on a hazardous cell. It's additionally crucial to consider that the particular traits of ways particularly specific antigens or epitopes purpose specific T-cellular responses are yet unknown [15]. The T-cell response is most likely coordinated towards numerous epitopes. The overall importance of response in opposition to a selected epitope is stochastic because of the responding T cells' international resistance for survival, initiation, arousal, and preparation [16]. Even as soon as spectator or vacationer T cells withinside the tumour microenvironment (TME) that do not notice malignant improvement epitopes are eliminated from consideration, this still appears to hold true. It is foreseeable with the capacity to differentiate among T-cell reactions in blood and malignancies. However, transient fluctuations consequently throw off such exams in person. Numerous immunogenic neo-antigens are present within the majority of contamination sufferers and might cause a T-cell response. The energy and profundity of cytotoxic-resistant reactions are probably enhanced through the breadth of these T-cell reactions. Despite this, intratumoral heterogeneity makes it more difficult to evaluate the significance of a given T-cell reaction in opposition to a particular neo-antigen. This is related to the stochastic notion of T-cell stimulation and obstruction observed inside the TME. The opposite of this is hostile to viral T-cell insusceptibility, in which a dominant clonal T-cell reaction against a specific viral epitope typically develops until that viral epitope is lost, at which point another dominant clonal T-cell reaction against another viral epitope may develop [11]

3. Modulating and predicting immune toxicity for better efficacy

Immunotherapies are frequently constrained by their Immune-Related Adverse Events (irAEs), an impervious actuation, and an incendiary reaction toward the host's healthy tissues. The desired outcome is resistant actuation against the host's development, although irAEs attempt to predict, examine, and treat [17]. The growth of a CTLA-4 immune response to PD-1 blockade in the context of metastatic melanoma is associated with a slow increase in endurance but at the cost of more than twice the rate of significant irAEs [18]. According to a recent meta-analysis, one casualty out of every 77 patients may receive an intracavernosal injection (ICI) mix treatment. The mortality rate is around 50% in patients who have received treatment for explicit irAEs, such as resistance-associated myocarditis. Numerous irAE markers have been put forth (such as measuring lymphopenia and eosinophilia, B cell alterations, T cell accumulation, circulating IL-17, and stomach microbiota changes). Still, only a small number have been partially validated [19].

According to guidelines, extensive immunosuppression with corticosteroids, followed by at least one biologic (growth factor receptor inhibitors) or T cell suppressor (such as mycophenolate mofetil), is recommended for significant irAEs [20]. Very little planned information has been developed about the outcomes of those treatments for products related to the disease. An analysis of the everyday use of corticosteroids in patients with lung cellular breakdown revealed a connection with worse endurance outcomes [21]. Additionally, more miserable survival was linked to high-portion steroids in patients with metastatic melanoma with resistant associated hypophysitis [22]. However, there was no correlation between using corticosteroids in other clinical situations where patients had irAEs and a reduced response to ICI treatment or endurance. To avoid impairing the effectiveness of ICIs, more studies are anticipated to examine the best immunosuppressive regimen to be used with them. In the specific context of organ transplantation, the use of mechanistic target of rapamycin (mTOR) inhibitors demonstrates a commitment to lowering toxic levels without impairing ICI viability [23].

4. Cost-effectiveness

Previous studies have proven that the cost-effectiveness of immunotherapy varies, generally relying on the therapeutic signs and the usage of biomarkers, along with modified passing ligand one status. The abundance of patients suffering is decreasing, and development is made in situations like NSCLC, wherein biomarkers are used for narrowing down the patient selection. In this approach, immunotherapy may be financially realistic in spite of its excessive price. Given the fair, distinctive endurance benefit of pembrolizumab in KEYNOTE-240, [20] it can now no longer come as a shock that the drug has been altered to currently being no longer cost-effective, even on the better cap of $300,000 for every quality-changing life-year [24]. However, in keeping with KEYNOTE-240, 19% of sufferers who dealt with pembrolizumab have been capable of holding their motion-free status for more than a year. In a disease with a usually brief survival time (10.7 months in 2008), this supported benefit within the minority is noteworthy. It offers patients with hepatocellular carcinoma renewed hope to live longer [25].

This additionally consists of the restrictions of cost-viability research for immunotherapies, which generally produce inconsistent consequences, with a small percent of patients experiencing more widespread advantages than the average. These drugs, in major opinion, are more significant to society than those that produce additional homogenous outcomes with a comparable low-middle endurance advantage. Oncologists are supported in a career, that is so full of distress, with the aid of memories of patients who overcame the adversity [26]. When weighing the prices and advantages of a drug like pembrolizumab, we recognise a critical question: what is the ideal balance between expensive treatments that benefit a minority and the overall financial stability of the system? [27].

5. The tumour suppressive microenvironment

There is sufficient evidence from experimental models and human subjects to demonstrate that persistent antigen exposure in the growth microenvironment causes CD8+ T lymphocytes to become depleted or damaged (TME). These damaged/depleted T cells exhibit distorted cytokine production and proliferative limitations. They appear capable of using lytic functions and are, thus, not wholly idle [28]. Broken CD8+ T cells up-regulate several inhibitory receptors (IRs)/safe, designated sites, such as PD-1, CTLA-4, T cell immunoglobulin and mucin domain-containing-3 (Tim-3), lymphocyte activation gene 3 (LAG-3), B- and T-lymphocyte attenuator (BTLA), and T cell immunoreceptor with Ig and immunoreceptor tyrosine-based inhibitory motif (ITIM) domains (TIGIT), that tight spot to their ligands transmitted by cancer cells and antigen-presenting cells (APCs) in the TME [29]. As a result, double safe designated spot blockade appears to more readily boost T-cell function and development and advance growth dismissal both in vitro and in vivo. The FDA-supported double CTLA-4/PD-1 barricade's recent progress against advanced melanoma disease emphasises the therapeutic validity of such a strategy [30].

Even though CD8+ tumour‑infiltrating lymphocytes (TILs) in the TME seem to up-regulate IRs, they also appear to do so for several enacting receptors (ARs), including 4-1BB, OX40, and glucocorticoid-induced tumour necrosis factor receptor (TNFR)-related protein (GITR) [31]. These are members of the TNFR family who, upon ligation, can rapidly co-invigorate T-cell capabilities. In preliminary clinical studies, antagonist monoclonal antibodies have shown promising therapeutic effects against malignant growth mice models. These agonist specialists work in synergy with designated spot inhibitors in preclinical animals [32].

**Figure 1:**
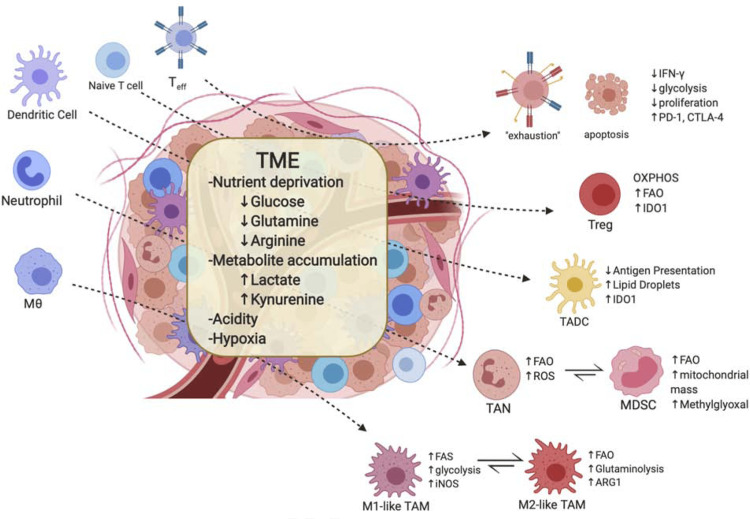
Tumour microenvironment (TME) and associated immune cell metabolism Teff- effector T cells; MO- macrophages; IF-gamma- interferon gamma; Pd-1- programmed cell death protein 1; CTLA-4- cytotoxic T-lymphocyte-associated antigen 4; Treg- regulatory T cells; Oxphos- oxidative phosphorylation; FAO- fatty acid oxidation; IDO-1- indoleamine dioxygenase; TADC- tumour-associated dendritic cells; TAN- tumour-associated neutrophils; ROS- reactive oxygen species; MDSC- myeloid-derived suppressor cells; M1- anti-tumour macrophages (M1 type); M2- anti-tumour macrophages (M2 type); TAM- tumour-associated macrophages; iNOS- inducible nitric oxide synthase; ARG-1- arginase 1 Image source: Bader et al., 2020 [36] (Open Archive). This is a non-commercial use of the image.

Determining among cancer patients who will respond to immunotherapies that target immunoregulatory pathways and choosing when different systems may be predicted to trigger T-cell responses to malignancies are essential questions [33]. The answer to this query may be found in the metastatic melanoma quality mark research, which proposes categorising tumours into "aggravated" and "non-aggravated" phenotypes [34]. Non-excited cancers require cancer-penetrating T cells and may need to be treated with novel targeted therapies (sting agonists, inhibitors of the catenin pathway) to encourage T-cell activation and movement into the growths. While aroused gains are immediately immunogenic and may be bound to respond to safe intercessions to neutralise the tools of cancer-initiated T-cell brokenness, non-excited cancers do not [35]. Figure 1 depicts the association of TME with immune cell metabolism about how the TME is often characteristic of various conditions including nutrient competition, low pH, limited oxygen, etc. and how such conditions result in immunosuppressive or tolerogenic phenotypes in general.

## Conclusions

Disease immunotherapy has significantly improved patients' endurance and sense of fulfilment. In any event, not all tumours are created equal, and there aren't many warning signs of toxicity yet. Despite the rapid advancements made in the area, immuno-oncology is still in its relative infancy and faces many challenges and roadblocks that must yet be overcome. After some time, it was recognised that the traditional methods used to evaluate drug choices during the age of chemotherapy and specific treatments certainly wouldn't be appropriate for the new immunotherapies. Extending the viability of combination treatments established in clinical practice is becoming increasingly challenging. Few innovative mixes have achieved a level of viability matching those new standards of care in the vast scene of ongoing starting-stage clinical preliminary studies. Their security profiles need to be upgraded, without a doubt. 

Immunotherapy goals are a one-of-a-kind backwater in most cancer remedies that have begun to expose promise of seeing their essential conceptualisation. However, the response percentage keeps varying for indefinite reasons after being tested from many viewpoints, with impervious technique and variation, variable antigen particularity and articulation levels, and very recently, the function through the gut microbiota. As a result, the complexity of the insusceptible shape and the elements contributing to its movement aren't sufficiently depicted, and similar research would require transdisciplinary techniques.
